# Interindividual Variation in the Relationship of Different Intensity Markers—A Challenge for Targeted Training Prescriptions

**DOI:** 10.1371/journal.pone.0165010

**Published:** 2016-10-27

**Authors:** Florian Egger, Tim Meyer, Anne Hecksteden

**Affiliations:** Saarland University, Institute of Sports and Preventive Medicine, Saarbrücken, Germany; Universidade de Tras-os-Montes e Alto Douro, PORTUGAL

## Abstract

**Purpose:**

Training intensities are frequently prescribed as relative workloads based on a single reference value (e.g. maximum oxygen uptake). However, exercise-induced physical strain is multifaceted and large interindividual variability in intensity markers has been reported for constant load exercise with standardized relative intensity. This questions the accuracy of (univariate) relative intensities in targeting specific training stimuli. The present trial aims to investigate interindividual variability in the relationship of strain indicators using interpolated performance curves derived from constant load tests at different workloads. This approach enables the prediction of other indicators based on a chosen reference and subsequent comparison of predictive accuracy between group-based and individualized regression models.

**Methods:**

15 competitive cyclists completed a stepwise incremental cycling test followed by 5 constant load tests with the same absolute workloads as in the stepwise incremental test. The highest of theses workloads which yielded a lactate (BLa) steady state was repeated enabling estimation of intraindividual variability. From constant load tests, the courses of BLa relative to the respective reference value (e.g. %VO_2peak_) were interpolated by polynomial regression. Variability between individual regression curves was analyzed by mixed modeling. Predictive accuracy was estimated as the sum of squared differences between predicted and observed values.

**Results:**

The proportion of total variation in the course of BLa relative to the respective reference parameter accounted for by subject identity ranged between 36 and 51%. A significant increase in predictive accuracy was observed for VO_2peak_ and HR_max_, respectively, as predicting parameters.

**Conclusion:**

These results are in support of a multivariable, individualized approach to intensity prescriptions when aiming at accurately targeted perturbations of homeostasis.

## Introduction

The aim of prescribing a specific training intensity is to induce a stimulus for adaptation as precisely targeted and standardized as possible. The kind and magnitude of this stimulus is only indirectly determined by absolute training load (e.g. power output or speed). Rather, the exercise-induced disturbances of homeostasis are the starting points of signaling processes, which finally link physical exercise to adaptations [1,2]. Beyond this mechanistic role in the generation of training effects, exercise-induced physical strain reflects the localisation of absolute exercise intensity on the continuum between resting level and maximal performance capacity of the individual. Therefore, training intensities are typically prescribed as relative intensities based on a reference value measured in the individual [1]. Practically important examples of such reference values are maximum oxygen uptake (VO_2max_ or VO_2peak_), maximum heart rate (HR_max_), their respective reserves or various other surrogate indicators (e.g. lactate and respiratory thresholds) of endurance capacity [3,4]. Importantly, exercise-induced strain is multifaceted encompassing cardiopulmonary, metabolic and physically defined domains on the cellular as well as on the systemic level. Therefore, characterizing a state of exercise-induced physical strain intrinsically calls for a multivariate approach. Despite this consideration, training intensities are generally prescribed relative to a single reference value mainly for practical reasons. This bases on the assumption that the relationship between different intensity markers is constant e.g. 60% VO_2max_ corresponds to 80% HR_max_ and 2 mmol·l^-1^ blood lactate concentration (BLa) [5]. However, markedly heterogeneous BLa and HR responses have been reported for constant load tests at fixed percentages of VO_2max_ [5,6]. These results question the assumption of fixed relationships between different intensity domains [1] and cause concern about the performance of a single reference value in terms of accurately standardized training intensities.

Yet, it has to be kept in mind that the design of the available seminal trials [5,6] is associated with several limitations:

1. Technical measurement error and day-by-day biological variability limit the accuracy of measurement for any reference value (e.g. VO_2max_). By using relative intensity prescriptions, this inaccuracy will inevitably be transmitted to the setting of the constant load tests. The resulting limited standardization of the independent variable (preset exercise intensity) will inflate observed variability in dependent markers. The magnitude of this effect is unstudied so far.
2. Intraindividual variability in the responses of the dependent markers is not assessed. This further limits inferences about the magnitude of true interindividual variation [7]. This aspect is particularly relevant because the proportion of interindividual variation determines the prospect of individualized approaches. Moreover, modifiable moderators (e.g. carbohydrate intake for BLa responses) let a substantial intraindividual variation seem plausible.
3. Constant load tests at different relative intensities are analyzed separately [5,6] leading to pointwise estimates of interindividual variability in strain markers at one specific relative intensity. By contrast, information about performance-related courses over the intensity spectrum of aerobic endurance exercise is missing. However, such information would be crucial for the generation of individualized prediction models of exercise induced strain.

Therefore, the main aim of this study was to quantify interindividual variation in the relationships between the load-dependent courses of different intensity markers. Moreover, the potential gain in predictive accuracy by considering this variability in individualized regression models was estimated. For this pilot work, BLa has been chosen as the predicted variable representing the relationship between cardiocirculatory and metabolic strain. Indicators of cardiopulmonary strain are used as predictors. Three design features were implemented addressing the above mentioned limitations:

1. Absolute intensities are used during constant load tests leading to high accuracy and precision in preset intensities.
2. Partial repetition enables estimation of intraindividual variability.
3. Mean values from constant load tests at different intensities are interpolated to obtain load-dependent courses. Analogously to incremental exercise tests, the commonly used method of creating lactate performance curves by polynomial regression was applied.

## Methods

### General design

Subjects performed 1 stepwise incremental cycling test to exhaustion followed by 5 constant load tests with absolute intensities identical to the steps of the incremental test (ensuring accurate standardization of preset intensities cp. above). The constant load test with the highest workload below the maximum lactate steady state (MLSS) [8] was repeated to enable separation of between- and within-subject variance components [7]. Constant load tests were conducted in random order, single blinded, separated by at least 48 hours of rest and at the same time of day. All tests for a specific subject were completed within five weeks.

**Fig 1:**
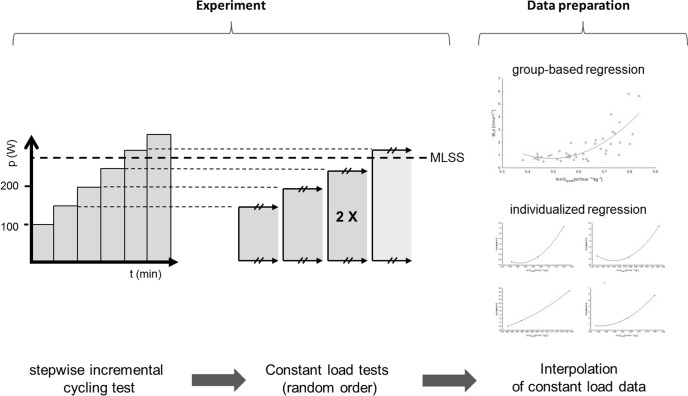
General Design. Subjects performed 1 stepwise incremental cycling test to exhaustion followed by 5 constant load tests with absolute intensities identical to the steps of the incremental test. The constant load test with the highest workload below the maximum lactate steady state (MLSS) was repeated. In analogy to the performance curves routinely generated from stepwise incremental exercise tests, load dependent courses were deduced from measurements during the constant load tests. Group-based as well as individual regression was implemented to assess potential increases in predictive accuracy with individualization. P (W) = Performance in watt; t (min) = time in minutes.

Load dependent courses of BLa relative to other strain markers were deduced by polynomial regression using the means of measured values from the respective constant load tests. This is in analogy to the lactate performance curves routinely interpolated from stepwise incremental exercise tests. Regression was performed in a group based fashion as well as separately for each athlete. Predictive accuracy of the resulting uniform or individualized regression equations, respectively, was then assessed as the agreement between predicted and observed values of BLa for the repeated constant load test. The proportion of interindividual differences in total variability was quantified by mixed modeling. An overview over general design features is given in Fig 1. A more detailed description of employed data analysis techniques can be found below.

### Subjects

15 trained male athletes (6 road cyclists, 5 mountain bikers, 4 triathletes; VO_2peak_ 67 ± 7 ml·kg^-1^·min^-1^, age 25 ± 4 years, height 180 ± 5 cm, weight 74 ± 7 kg) volunteered to participate in this investigation. Individuals were eligible if they fulfilled the following inclusion criteria: male, age 18–35 years, individual anaerobic threshold (IAT) ≥ 200 W corresponding to an oxygen uptake of ≥ 50 ml·kg^-1^·min^-1^. All subjects were over 18 years of age, were fully informed about the experimental procedures and gave written informed consent prior to participation. The study was carried out in accordance with the declaration of Helsinki and approved by the local ethics committee (Ärztekammer des Saarlandes, Saarbrücken, Germany; approval number: 23/14); see S1 and S2 Protocols. In addition, all relevant data are presented in tabular form; see S1 Data.

### Outcome measures

From the multitude of indicators for exercise induced physical strain, practically relevant measures of training intensity were selected for this analysis. A particular focus was on the relationship between cardiocirculatory parameters routinely used for training prescription and BLa. The following predictors were included: % of maximal heart rate (%HR_max_), % of peak oxygen uptake (%VO_2peak_), and the respective reserves (%HR_R_; %VO_2R_).

### Exercise testing procedures

Subjects were advised to abstain from exercise (>30 min), alcohol and caffeine 48 h prior to testing and to maintain their usual carbohydrate-rich diet. Prior to each test, subjects received standardized questions regarding self-evaluation of physical condition, hydration status and compliance to dietary and exercise restrictions. Subjects performed all trials with clipless pedals on their own bikes attached to an electromagnetically bicycle ergometer (Cyclus 2, RBM elektronik-automation GmbH, Leipzig, Germany). Individual seat and handlebar position was maintained throughout.

### Incremental exercise test

All subjects started cycling at 100 W and workload was increased every 3 min by 50 W until exhaustion. Arterialized capillary blood was obtained from the hyperemezid earlobe for analyzing BLa (enzymatic-amperometric method, Greiner, Flacht, Germany) at the end of each step, at cessation and 1, 3, 5, 7 and 10 min post-exercise. Based on BLa performance curve IAT was determined using previous methods by Stegman et al. [8]. Gas exchange parameters were measured continuously with a MetaMax II metabolic test system (Cortex Biophysik, Leipzig, Germany; mixing chamber; sampling frequency 10 s). The mean of the 3 highest values of VO_2_ was defined as peak oxygen uptake (VO_2peak_). HR_max_ was derived from continuous ECG recordings.

#### Constant load tests

Workloads for the constant load tests were the following: 3 highest absolute workloads of the stepwise incremental test below IAT, 1 repetition test for the highest of these workloads, 1 test with the workload following IAT to confirm estimation of MLSS by the absence of lactate-steady-state conditions. The target duration of constant load tests was 60 min. Test sequence of constant load tests was randomized and subjects were not informed about the workload applied during a specific test.

After completing a 5 min warm-up session at 100 W, workload was adjusted as randomized for the respective test and subjects continued cycling for 60 min. BLa was determined every 10 min and gas exchange measurements were carried out continuously. Means of the measured values for the last 50 min of the constant load test were taken for further analysis. VO_2R_ and HR_R_ were calculated as difference between the highest values, VO_2peak_ and HR_max_ respectively, and resting values. Lactate steady state was defined according to Beneke et al. as an increase of BLa of < 1 mmol·l^-1^ between the tenth exercise minute and cessation of exercise [9].

### Statistics

A main aim of this trial was the quantification of variability in the dependent variables. For this case, meaningful a priori sample size calculations were not feasible. Therefore, sample size was chosen according to a minimum detectable effect size of 0.8 for the between-approach comparison in the agreement between predicted and observed values.

All analyses were performed with the Statistica 10 software package (Statsoft, Tulsa, Oklahoma, USA). Normal distribution was verified using the Shapiro-Wilks W-test. Consequently, parametrical methods were applied throughout. Descriptive data are presented as means ± standard deviation (SD). The significance level for the alpha error was set at p<0.05.

#### Generation of load-dependent courses

Mean values of BLa from the three constant load tests with different intensities were interpolated to generate load dependent courses. This proceeding mimics the routine deduction of performance curves from stepwise incremental exercise tests. As depicted in Fig 1, regression equations were fit in a group-based approach as well as separately for each individual to enable comparisons between group-based and individualized prediction of the respective dependent parameter.

#### Interindividual variability in the relationship between load dependent courses

Interindividual variability in the relationship between the courses of a dependent strain indicator relative to a (predicting) reference was analyzed by mixed modeling (random effect: subject identity; covariate: reference parameter; restricted maximum likelihood estimation). Due to the non-linear behavior of the BLa performance curve a polynomial (quadratic) model was employed. All four constant load tests below MLSS were included in this analysis. The repetition test enables estimation of intraindividual variability and avoids overestimation of interindividual differences [7].

#### Potential increase in predictive accuracy with individualized prediction

Observed BLa mean values for the respective reference parameter from the repeated constant load tests were entered into group-based and individual regression equations. The equations were derived as described above, with the exception that the repeated constant load test was not included. Subsequently, the resulting predicted values for the dependent variables were compared to the observed values in the same individual. The sum of squared differences (SSD) between predicted and observed values was used as estimate of predictive accuracy. The % difference in SSD was used to quantify the increase in predictive accuracy with individualized prediction as compared to the group-based approach. Moreover, agreement between predicted and observed values is expressed as limits of agreement [10].

## Results

All participants finished the study. Peak oxygen uptake and maximum workload achieved during incremental exercise tests were 67 ± 7 ml·kg^-1^·min^-1^ and 5.2 ± 0.5 W·kg^-1^, respectively. Objective criteria of exhaustion (HR_max_ > 200 (min^-1^) minus age (years), BLa_max_ > 8 mmol·l^-1^; RQ_max_ > 1.1) were fulfilled for all stepwise incremental tests by each subject. Workload at IAT was 281 ± 36 W (3.8 ± 0.5 W ·kg^-1^). Correct estimation of MLSS could be confirmed during constant load tests for all subjects by the presence of lactate steady state at the highest workload below IAT and the absence of lactate steady state conditions at the workload above IAT. According to IAT the following workloads applied for constant load tests aiming at steady state conditions: In five cases: 300 W, 250 W and 200 W; in seven cases: 250 W, 200 W and 150 W; in three cases: 200 W, 150 W and 100 W.

### Variance components

The proportion of total variance accounted for by subject identity (between-subject variation) ranged between 36 and 51% depending on the predicting parameter (Table 1).

**Table 1:** Interindividual variation and predictive accuracy.

Independent variable	Interindividual variation		Predictive accuracy	
	% total variance	total*	change with individualized prediction ^#^	
%VO_2peak_	36	1,00	31	**0.01**
%VO_2R_	51	1,44	29	0.42
%HR_max_	40	0,74	75	**0.04**
%HR_R_	44	0,84	81	0.09

Dependent variable in all cases: blood lactate concentration

SD: Standard deviation, %: percentage of, HR_max_: maximum heart rate, HR_R_: heart rate reserve, VO_2peak_: peak oxygen uptake, VO_2R_: oxygen uptake reserve.

* As SD [mmol·l^-1^]

^#^ The sum of squared differences between predicted and observed values for the repeated constant load test is used as estimate of predictive accuracy. The increase in predictive accuracy with individualized prediction is quantified as % decrease in sum of squared differences. The significance level for the alpha error was set at p<0.05.

### Agreement between predicted and observed values

**Fig 2:**
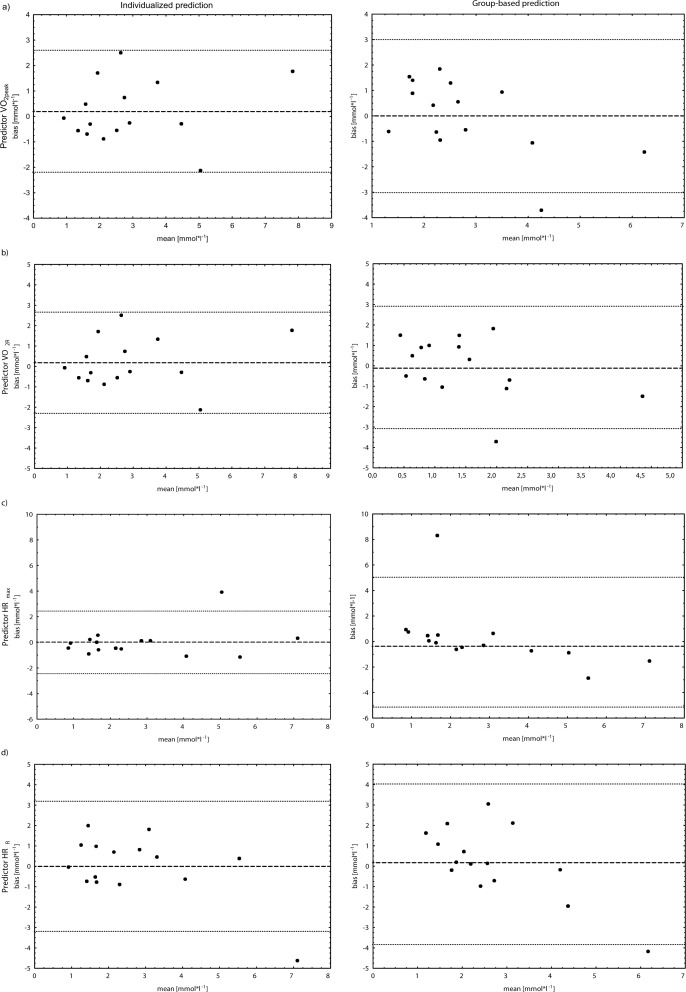
Predictive accuracy. Agreement between predicted and observed blood lactate concentrations [BLa] for individualized and group-based prediction is expressed as limits of agreement [10]. Predictors: % of maximal heart rate (%HR_max_), % of peak oxygen uptake (%VO_2peak_) and the respective reserves (%HR_R_; %VO_2R_). Bias = bias of BLa observed and predicted; mean = mean of BLa observed and predicted; spotted line = mean bias; dashed line = limits of agreement.

Sums of squared differences (SSD) between predicted and observed values for the repeated constant load test were used as estimates of predictive accuracy. A numerical decrease in SSD with individualized prediction as compared to the group-based approach could be observed for all predictors (Table 1). For %HR_max_ and %VO_2peak_, respectively, the difference in SSD between the two approaches reached statistical significance. For the prediction of BLa using %HR_R_ as the predicting parameter a tendency towards significance was observed. Limits of agreement are presented in Fig 2.

## Discussion

In this trial considerable interindividual variability has been demonstrated for most parameters. Importantly, this is despite a trial design, which is focused on eliminating confounding sources of variability. Improvements in predictive accuracy with individualized regression equations further underline the potential practical relevance of an individualized, multivariate approach to prescribing exercise intensities (Fig 2).

Targeted and standardized training stimuli are the main aim of prescribing specific training intensities. However, while training intensities are usually based on one reference value [1] exercise induced physical strain involves a multitude of parameters many of which are starting points for the signaling cascades which mediate adaptive training responses [1,2]. Therefore, interindividual variation in the relationship between such parameters can theoretically impede on the accuracy and precision of prescriptions for training intensity.

The gap between the multifaceted character of exercise induced physical strain and univariate training intensities requires the (implicit) assumption of constant relationships between strain indicators [1]. Only if this assumption holds true, prescribing training intensity based on a single reference parameter will lead to standardized values for other strain indicators, too [1,2,5,7]. Despite the fundamental importance of targeted and standardized training stimuli for research and training practice, so far only one experimental trial has explicitly investigated this assumption [5]. The authors report large variability of heart rate and blood lactate responses for constant load tests at fixed percentages of VO_2max_ [5]. However, this practical design is associated with limitations regarding the quantification of true interindividual variability, which is decisive for the prospect of individualization [11]. Two aspects are of particular importance: (i) Measurement error in the reference value (VO_2max_ or VO_2_, respectively) will inevitably lead to imprecision in the independent variable during the constant load tests. (ii) Intraindividual variability in the relationship between oxygen uptake and the dependent parameters is likely to be present (e.g. due to differences in carbohydrate intake or fluid status) but cannot be estimated due to the lack of repeated testing [7]. Taken together the large variation observed in the trial by Scharhag-Rosenberger et.al. [5] strengthened initial doubts concerning negligible variation in the relationships between different strain indicators. However, the magnitude of true interindividual variation remained unknown. In the present trial absolute intensities and repeated testing of subjects have been implemented to estimate and (mathematically) eliminate the mentioned confounding sources of variability. The resulting estimates of interindividual variability differ between predicting parameters but generally point towards the superiority of individualized approaches if optimal accuracy of training prescriptions is intended.

While the magnitude of true between-subject variability is critical for the prospect of individualization, determining the potential increase in predictive accuracy requires quantification of agreement between predicted and observed values for both approaches (group-based and individualized). In the present trial, this kind of analysis was realized by using regression equations of interpolated “performance curves” together with the values of predicting and predicted parameters observed in the repeated constant load tests. The improvements in predictive accuracy realized by using individualized regression support adequacy and relevance of an individualized, multivariate approach. Moreover, individualized regression curves offer an intuitively interpretable implementation, analogous to the performance curves commonly used in performance diagnostics.

Based on the results of this trial, allowing for individual relationships between strain indicators in training prescription (e.g. by individualized regression equations) may contribute significantly to precisely targeted and standardized training prescriptions. However, it is important to keep in mind that this is foundation work aiming to establish proof-of-concept for a new look on training intensity rather than to develop practical implementations. Meaningful transfer into training practice requires detailed insights into the signaling and adaptive processes emanating from individual strain parameters as well as their interactions. In other words: target values and importance of different strain parameters for specific training goals have to be established. Unraveling these causal chains requires long term training trials with appropriate designs. Likewise, variability in other, namely intracellular, strain parameters and the activation of the depending signaling cascades remains to be investigated.

In some instances the individualized regression equation predicted BLa in the repetition test considerably worse as compared to the group-based approach. When inspecting data from these cases, either a markedly atypical behavior of the individual regression curve or a large deviation in BLa between the original and the repetition test could be seen. As three data points are always fit perfectly by a quadratic polynomial, it may be argued that this is caused by random variation in opposite directions within the small number of observations per individual. This consideration points to a major downside of individualized predictions: the smaller number of observations leads to a higher impact of random variation. A practicable solution to this dilemma, in particular when transfer into training practice is attempted, could be the updating of regression curves by inclusion of data from constant load training bouts.

The repeated constant load test has been included in the design of this trial to enable estimation of: (i) intraindividual variability, and (ii) agreement between predicted and observed values for group-based and individualized regression. In particular with respect to (i) repetition of all three constant load tests would have been preferable. However, the additional testing burden was not acceptable for the competitive athletes enrolled in this trial. In consideration of this limitation and to avoid overestimation of between-subject variability, the highest workload still yielding a lactate steady state has been chosen for repetition. This decision was based on previous research pointing to higher variation with higher intensities [5].

## Conclusion

Interindividual variability of relationships between indicators of exercise induced physical strain is substantial. Taking these individual differences into account, e.g. by individualized regression curves, can lead to more accurate estimates of dependent strain indicators and therefore to a better characterization of training stimuli. These results point to the superiority of an individualized, multivariate approach to prescribing exercise intensities if optimal accuracy is intended. Longitudinal training trials are warranted to examine potential improvements in exercise efficacy with the shown individualized approach.

## Supporting Information

S1 Data Primary data. (PDF)

S1 Protocol Trial study protocol approved by the local ethics committee (translated version). (PDF)

S2 Protocol Studienprotokoll anerkannt durch die lokale Ethikkommission (original language). (PDF)

## References

[1] Mann T, Lamberts RP, Lambert MI Methods of prescribing relative exercise intensity: physiological and practical considerations. Sports Med. 2013; 43: 613–625. 10.1007/s40279-013-0045-x 23620244

[2] Coffey VG, Hawley JA The molecular bases of training adaptation. Sports Med. 2007; 37: 737–763. 17722947 10.2165/00007256-200737090-00001

[3] Faude O, Kindermann W, Meyer T Lactate threshold concepts: how valid are they? Sports Med. 2009; 39: 469–490. 10.2165/00007256-200939060-00003 19453206

[4] Dekerle J, Baron B, Dupont L, Vanvelcenaher J, Pelayo P Maximal lactate steady state, respiratory compensation threshold and critical power. Eur J Appl Physiol. 2003; 89: 281–288. 10.1007/s00421-002-0786-y 12736836

[5] Scharhag-Rosenberger F, Meyer T, Gassler N, Faude O, Kindermann W Exercise at given percentages of VO2max: heterogeneous metabolic responses between individuals. J Sci Med Sport. 2010; 13: 74–79. 10.1016/j.jsams.2008.12.626 19230766

[6] Meyer T, Gabriel HH, Kindermann W Is determination of exercise intensities as percentages of VO2max or HRmax adequate? Med Sci Sports Exerc. 1999; 31: 1342–1345. 10487378 10.1097/00005768-199909000-00017

[7] Hecksteden A, Kraushaar J, Scharhag-Rosenberger F, Theisen D, Senn S, Meyer T Individual response to exercise training—a statistical perspective. J Appl Physiol. 2015; 118: 1450–1459. 10.1152/japplphysiol.00714.2014 25663672

[8] Stegmann H, Kindermann W, Schnabel A Lactate kinetics and individual anaerobic threshold. Int J Sports Med. 1981; 2: 160–165. 10.1055/s-2008-1034604 7333753

[9] Beneke R Methodological aspects of maximal lactate steady state-implications for performance testing. Eur J Appl Physiol. 2003; 89: 95–99. 10.1007/s00421-002-0783-1 12627312

[10] Bland JM, Altman DG Statistical methods for assessing agreement between two methods of clinical measurement. Lancet. 1986; 1: 307–310. 2868172

[11] Senn S, Rolfe K, Julious SA Investigating variability in patient response to treatment—a case study from a replicate cross-over study. Stat Methods Med Res. 2011; 20: 657–666. 10.1177/0962280210379174 20739334

