# Supplementary material for: Interindividual Variation in the Relationship of Different Intensity Markers—A Challenge for Targeted Training Prescriptions
Source: PLoS One. 2016 Oct 27;11(10):e0165010. doi: 10.1371/journal.pone.0165010 (PMC5082935; doi:10.1371/journal.pone.0165010)
Supplement: S1 Data — (PDF) [file pone.0165010.s001.pdf]

Baseline characteristics

n	type of sport rb=1, mb=2, tri=3	age [y]	height [cm]	weight [kg]	BMI [kg/m^2]	VO_{2peak} [ml·min^{-1}·kg^{-1}]
1	2	31	188	78.5	22.2	67.4
2	1	27	183	87.9	26.2	56.0
3	2	23	179	71.2	22.2	71.5
4	3	33	184	73.1	21.6	59.4
5	2	25	173	70.9	23.7	65.0
6	3	26	183	87.6	26.2	67.0
7	2	25	189	81.6	22.8	66.5
8	3	24	178	70.4	22.2	76.0
9	1	26	172	65.5	22.1	67.7
10	1	24	183	72.4	21.6	67.7
11	2	18	173	62.9	21.0	79.9
12	1	27	179	71.0	22.2	60.8
13	1	25	182	70.0	21.1	56.7
14	3	21	180	72.8	22.5	73.4
15	1	25	183	70.0	20.9	68.4

rb = racing bike; mb = mountainbike; tri = triathlon

Incremental exercise test

n	HR_{rest} [bpm]	HR_{max} [bpm]	HR_R [bpm]	P_{max} [W]	P_{max} [$W \cdot kg^{-1}$]	VO_{2peak} [$ml \cdot min^{-1}$]	VO_{2peak} [$ml \cdot min^{-1} \cdot kg^{-1}$]	BLa_{rest} [$mmol \cdot l^{-1}$]	BLa_{max} [$mmol \cdot l^{-1}$]	IAT [$W \cdot kg^{-1}$]	IAT [W]
1	72	185	113	425	5.3	5301	67.1	0.8	12.9	4.2	330
2	64	198	134	392	4.5	4824	56.0	0.6	13.9	3.2	285
3	92	202	110	417	5.7	5178	71.5	0.9	14.3	4.2	298
4	85	186	101	400	5.1	4358	59.4	0.9	14.2	3.9	286
5	57	188	131	375	5.2	4697	65.0	1.1	10.9	4.0	284
6	56	188	132	450	5.1	5857	67.0	0.5	9.8	3.7	328
7	67	180	113	425	5.3	5355	66.5	1.3	14.3	3.6	295
8	52	187	135	408	5.7	5375	76.0	1.0	14.0	4.2	297
9	74	188	114	350	5.3	4454	67.7	0.7	13.3	3.4	224
10	50	182	132	400	5.5	4881	67.7	0.8	13.8	4.0	293
11	61	196	135	375	6.0	5019	79.9	0.8	11.3	4.5	283
12	62	190	128	325	4.6	4301	60.8	0.6	13.4	3.2	224
13	107	218	111	283	4.0	3972	56.7	0.8	14.4	2.9	203
14	85	186	101	400	5.4	5396	73.4	0.6	15.5	4.1	300
15	72	191	119	191	5.5	4744	68.4	0.6	14.4	4.1	285

HR_{rest} = heart rate at rest; HR_{max} = maximum heart rate; HR_R = heart rate reserve; P_{max} = maximum performance; VO_{2peak} = peak oxygen consumption
BLa_{rest}= blood lactate concentration at rest; BLa_{max}= blood lactate concentration at exhaustion; IAT= individual anaerobic threshold

Constant load test (lowest workload below IAT)

IAT = individual anaerobic threshold

n	workload [W]	HR_{rest} [bpm]	HR 10min [bpm]	HR 20min [bpm]	HR 30min [bpm]	HR 40min [bpm]	HR 50min [bpm]	HR 60min [bpm]	HR_{mean} [bpm]	% HR_{max} [%]
1	200	62	126	125	126	129	133	129	128	69
2	150	53	100	102	101	102	102	102	102	51
3	150	63	120	116	119	120	121	118	119	59
4	150	82	111	110	109	113	113	115	112	60
5	150	61	120	116	114	116	112	114	115	61
6	200	61	120	120	115	115	115	116	117	62
7	200	64	117	120	121	123	124	128	122	68
8	150	58	115	121	116	118	116	120	118	63
9	100	62	97	95	95	90	91	95	94	50
10	200	53	121	126	131	127	125	127	126	69
11	150	56	113	114	116	113	113	114	114	58
12	100	72	102	103	106	103	104	108	104	55
13	100	88	142	144	140	140	141	143	142	65
14	200	63	122	120	116	118	121	122	120	64
15	150	75	121	117	117	118	119	119	119	62

HR_{rest} = heart rate at rest; HR 10min-60min = heart rate at respective time; HR_{mean} = mean heart rate

$\%HR_{max}$ = percentage of maximum heart rate

Constant load test (lowest workload below IAT)

IAT = individual anaerobic threshold

n	BLa_{rest} [mmol·l^{-1}]	BLa 10min [mmol·l^{-1}]	BLa 20min [mmol·l^{-1}]	BLa 30min [mmol·l^{-1}]	BLa 40min [mmol·l^{-1}]	BLa 50min [mmol·l^{-1}]	BLa 60min [mmol·l^{-1}]	BLa_{mean} [mmol·l^{-1}]
1	0.9	0.6	0.6	0.6	0.6	0.5	0.5	0.6
2	0.7	1.2	0.8	1.1	1.0	0.7	0.5	0.9
3	0.9	1.2	1.0	1.3	1.2	1.1	1.1	1.1
4	1.2	1.0	1.0	0.9	1.1	1.3	0.6	1.0
5	1.0	1.5	1.1	0.7	0.8	1.1	0.6	1.0
6	1.0	0.7	1.0	1.0	1.2	1.0	1.2	1.0
7	0.7	0.9	0.7	0.5	0.7	0.7	0.5	0.7
8	1.1	1.1	0.9	0.8	0.8	0.9	1.0	0.9
9	0.7	0.5	0.6	0.7	0.7	0.7	0.6	0.6
10	0.9	1.1	1.2	1.0	0.8	0.7	0.6	0.9
11	0.7	0.5	0.5	1.3	0.5	0.6	0.6	0.7
12	1.0	0.8	0.9	0.9	0.8	0.6	0.8	0.8
13	0.8	1.0	0.8	0.6	0.7	0.7	0.6	0.7
14	1.3	0.7	1.0	0.7	0.8	0.7	1.0	0.8
15	0.7	0.6	0.5	0.5	0.5	0.5	0.6	0.5

BLa_{rest} = blood lactate concentration at rest; BLa 10min-60min = blood lactate concentration at respective time; BLa_{mean} = mean blood lactate concentration

Constant load test (lowest workload below IAT)

IAT = individual anaerobic threshold

n	VO_{2rest} [ml·min^{-1}·kg^{-1}]	VO_2 10min [ml·min^{-1}·kg^{-1}]	VO_2 20min [ml·min^{-1}·kg^{-1}]	VO_2 30min [ml·min^{-1}·kg^{-1}]	VO_2 40min [ml·min^{-1}·kg^{-1}]	VO_2 50min [ml·min^{-1}·kg^{-1}]	VO_2 60min [ml·min^{-1}·kg^{-1}]	VO_{2mean} [ml·min^{-1}·kg^{-1}]	%VO_{2peak} [ml·min^{-1}·kg^{-1}]
1	8.8	38.8	36.7	35.8	36.7	31.2	33.5	35.4	53
2	6.6	25.9	26.2	24.2	24.7	25.9	25.1	25.3	45
3	5.8	31.5	32.1	33.0	30.6	29.2	31.0	31.2	44
4	6.9	27.7	28.8	27.3	28.9	30.4	28.2	28.6	48
5	7.5	37.8	37.6	36.4	36.1	35.5	35.8	36.5	56
6	5.3	34.1	32.8	33.8	33.9	31.8	31.0	32.9	49
7	9.5	34.1	34.2	33.2	33.3	34.6	35.4	34.1	51
8	6.5	34.5	35.6	32.8	32.4	31.2	33.2	33.3	44
9	7.7	26.2	28.2	27.1	24.3	23.7	24.3	25.6	38
10	7.1	38.1	39.1	43.1	40.4	40.5	37.9	39.9	59
11	7.8	34.5	37.0	34.2	36.2	34.2	35.0	35.2	44
12	6.1	26.1	26.9	25.8	24.6	26.4	25.2	25.8	42
13	4.4	25.6	24.9	24.8	24.5	22.2	25.6	24.6	43
14	7.8	41.9	40.3	41.0	41.8	42.9	39.7	41.3	56
15	7.8	30.1	30.5	32.0	32.9	32.2	30.9	31.4	46

VO_{2rest} = oxygen consumption at rest; VO_2 10min-60min = oxygen consumption at respective time; VO_{2mean} = mean oxygen consumption; %VO_{2peak} = percentage of peak oxygen consumption

Constant load test (medium workload below IAT)

IAT = individual anaerobic threshold

n	workload [W]	HR_{rest} [bpm]	HR 10min [bpm]	HR 20min [bpm]	HR 30min [bpm]	HR 40min [bpm]	HR 50min [bpm]	HR 60min [bpm]	HR_{mean} [bpm]	% HR_{max} [%]
1	250	69	146	142	141	138	147	144	143	77
2	200	57	119	125	132	132	132	134	129	65
3	200	78	130	134	134	132	137	134	134	66
4	200	84	126	131	128	132	134	130	130	70
5	200	51	132	131	130	130	131	131	131	70
6	250	60	131	131	130	130	130	131	131	69
7	250	61	142	146	146	147	150	149	147	81
8	200	62	126	128	130	131	134	133	130	70
9	150	56	142	141	141	141	141	140	141	75
10	250	58	142	142	142	143	145	143	143	78
11	200	55	130	130	130	131	131	133	131	67
12	150	82	121	122	127	128	132	132	127	67
13	150	102	158	159	163	159	162	161	160	74
14	250	75	138	142	141	140	139	141	140	75
15	200	82	142	139	141	141	143	146	142	74

HR_{rest} = heart rate at rest; HR 10min-60min = heart rate at respective time; HR_{mean} = mean heart rate
%HR_{max} = percentage of maximum heart rate

Constant load test (medium workload below IAT)

IAT = individual anaerobic threshold

n	BLa_{rest} [mmol·l^{-1}]	BLa 10min [mmol·l^{-1}]	BLa 20min [mmol·l^{-1}]	BLa 30min [mmol·l^{-1}]	BLa 40min [mmol·l^{-1}]	BLa 50min [mmol·l^{-1}]	BLa 60min [mmol·l^{-1}]	BLa_{mean} [mmol·l^{-1}]
1	0.7	0.7	0.7	0.6	0.6	0.7	0.7	0.7
2	0.8	2.4	2.0	2.2	2.1	2.0	1.2	2.0
3	1.0	0.6	1.1	1.0	1.3	1.3	0.9	1.0
4	0.7	1.3	1.3	1.6	1.0	0.6	0.9	1.1
5	0.8	0.8	1.0	0.8	0.5	0.6	0.5	0.7
6	0.6	1.1	1.2	1.0	0.9	0.8	1.0	1.0
7	1.7	3.7	2.3	1.7	2.2	1.9	1.5	2.2
8	1.3	0.8	0.8	0.9	0.9	1.0	0.9	0.9
9	1.8	1.8	1.5	1.3	1.2	1.2	1.2	1.4
10	0.8	1.8	1.3	1.6	1.2	1.3	1.1	1.4
11	0.7	0.6	0.5	0.5	0.7	0.6	0.5	0.6
12	0.7	1.0	1.1	1.1	1.3	1.2	1.1	1.1
13	1.2	2.3	2.9	2.2	1.6	1.7	1.4	2.0
14	1.2	1.1	1.4	1.2	0.9	0.9	1.0	1.1
15	0.9	0.8	0.7	0.7	0.7	0.8	0.7	0.7

BLa_{rest} = blood lactate concentration at rest; BLa 10min-60min = blood lactate concentration at respective time; BLa_{mean} = mean blood lactate concentration

Constant load test (medium workload below IAT)

IAT = individual anaerobic threshold

n	VO_{2rest} [ml·min^{-1}·kg^{-1}]	VO_2 10min [ml·min^{-1}·kg^{-1}]	VO_2 20min [ml·min^{-1}·kg^{-1}]	VO_2 30min [ml·min^{-1}·kg^{-1}]	VO_2 40min [ml·min^{-1}·kg^{-1}]	VO_2 50min [ml·min^{-1}·kg^{-1}]	VO_2 60min [ml·min^{-1}·kg^{-1}]	VO_{2mean} [ml·min^{-1}·kg^{-1}]	$\%VO_{2peak}$ [ml·min^{-1}·kg^{-1}]
1	8.6	42.7	37.2	39.1	41.2	34.7	38.7	38.9	58
2	9.7	31.0	32.3	32.6	33.3	27.6	34.3	31.8	57
3	7.7	39.3	43.1	38.8	41.1	41.7	35.8	40.0	56
4	6.3	35.5	34.9	37.1	34.0	32.5	34.3	34.7	58
5	8.0	42.5	40.6	38.8	39.9	40.5	38.2	40.1	62
6	7.6	39.5	37.5	40.4	37.2	34.4	37.7	37.8	56
7	8.8	42.9	42.1	43.9	45.1	46.1	45.4	44.2	67
8	7.7	46.1	41.7	41.7	39.6	40.2	42.1	41.9	55
9	7.8	36.2	35.9	34.5	36.7	34.5	34.1	35.3	52
10	6.9	45.6	46.7	47.8	48.3	48.2	50.1	47.8	71
11	6.5	50.8	52.5	50.9	50.7	48.5	50.6	50.7	63
12	6.1	31.6	31.9	33.7	33.8	34.0	35.4	33.4	55
13	5.7	32.6	35.0	35.6	35.9	37.1	36.9	35.5	63
14	6.7	50.6	45.3	43.9	44.8	43.3	43.7	45.3	62
15	7.1	42.6	43.2	43.1	43.5	43.7	41.8	43.0	63

VO_{2rest} = oxygen consumption at rest; VO_2 10min-60min = oxygen consumption at respective time; VO_{2mean} = mean oxygen consumption; $\%VO_{2peak}$ = percentage of peak oxygen consumption

Constant load test (highest workload below IAT)

IAT = individual anaerobic threshold

n	workload [W]	HR_{rest} [bpm]	HR 10min [bpm]	HR 20min [bpm]	HR 30min [bpm]	HR 40min [bpm]	HR 50min [bpm]	HR 60min [bpm]	HR_{mean} [bpm]	% HR_{max} [%]
1	300	71	157	157	158	157	157	158	157	85
2	250	70	161	169	177	180	184	186	176	89
3	250	89	155	151	161	165	164	165	160	79
4	250	81	157	154	156	157	161	161	158	85
5	250	60	148	152	151	154	157	157	153	81
6	300	78	153	159	160	164	161	163	160	85
7	300	50	153	159	164	168	170	170	164	91
8	250	59	141	144	148	148	151	153	148	79
9	200	61	147	150	151	155	154	155	152	81
10	300	51	166	167	169	169	173	174	170	93
11	250	66	164	157	158	161	161	160	160	82
12	200	71	143	148	154	157	161	159	154	81
13	200	103	193	198	201	202	200	199	199	91
14	300	68	159	160	166	163	164	162	162	87
15	250	81	162	166	169	171	173	174	169	89

HR_{rest} = heart rate at rest; HR 10min-60min = heart rate at respective time; HR_{mean} = mean heart rate
$\%HR_{max}$ = percentage of maximum heart rate

Constant load test (highest workload below IAT)

IAT = individual anaerobic threshold

n	BLa_{rest} [mmol·l^{-1}]	BLa 10min [mmol·l^{-1}]	BLa 20min [mmol·l^{-1}]	BLa 30min [mmol·l^{-1}]	BLa 40min [mmol·l^{-1}]	BLa 50min [mmol·l^{-1}]	BLa 60min [mmol·l^{-1}]	BLa_{mean} [mmol·l^{-1}]
1	1.1	1.6	1.2	1.0	1.1	1.0	1.0	1.1
2	1.2	4.0	3.3	3.9	4.8	4.5	5.0	4.2
3	0.8	1.9	2.1	2.2	1.8	1.5	1.9	1.9
4	0.9	4.7	2.2	2.7	2.6	2.2	2.1	2.7
5	1.0	1.0	0.9	1.0	1.0	1.3	1.1	1.0
6	1.2	3.0	3.1	2.6	2.5	3.0	3.0	2.9
7	1.1	4.0	3.7	3.7	3.9	3.3	3.0	3.6
8	0.9	2.1	2.0	2.1	1.7	1.8	1.8	1.9
9	1.1	2.4	2.7	2.4	2.2	2.1	2.1	2.3
10	1.0	5.5	5.9	5.4	6.0	6.6	4.5	5.7
11	0.8	2.1	1.6	1.6	1.4	1.2	1.2	1.5
12	1.3	1.9	1.9	2.0	2.2	2.1	1.8	2.0
13	0.7	6.9	7.1	5.6	5.7	5.9	3.9	5.8
14	1.3	2.7	3.1	3.6	2.4	2.5	1.7	2.7
15	0.9	1.6	1.7	1.9	1.8	2.1	2.4	1.9

BLa_{rest} = blood lactate concentration at rest; BLa 10min-60min = blood lactate concentration at respective time;
BLa_{mean} = mean blood lactate concentration

Constant load test (highest workload below IAT)

IAT = individual anaerobic threshold

n	VO_{2rest} [ml·min^{-1}·kg^{-1}]	VO_2 10min [ml·min^{-1}·kg^{-1}]	VO_2 20min [ml·min^{-1}·kg^{-1}]	VO_2 30min [ml·min^{-1}·kg^{-1}]	VO_2 40min [ml·min^{-1}·kg^{-1}]	VO_2 50min [ml·min^{-1}·kg^{-1}]	VO_2 60min [ml·min^{-1}·kg^{-1}]	VO_{2mean} [ml·min^{-1}·kg^{-1}]	%VO_{2peak} [ml·min^{-1}·kg^{-1}]
1	8.4	51.4	48.7	49.4	48.0	41.4	44.4	47.2	70
2	9.7	40.9	37.6	41.7	44.1	38.4	40.9	40.6	73
3	11.0	54.3	55.5	47.5	53.5	49.5	49.9	51.7	72
4	8.4	45.0	43.4	46.3	43.2	43.4	42.2	43.9	74
5	6.8	48.3	52.0	52.6	47.3	46.6	48.2	49.1	76
6	8.9	46.8	48.7	48.4	49.3	50.4	50.1	49.0	73
7	5.0	50.6	50.5	49.5	49.3	52.1	50.2	50.4	76
8	4.4	49.7	51.2	52.0	52.0	48.2	49.6	50.4	66
9	9.6	42.9	46.3	47.4	49.0	49.0	47.1	46.9	69
10	6.5	56.2	58.2	55.8	56.8	56.0	56.2	56.5	84
11	8.3	54.7	55.9	56.0	53.2	54.8	53.5	54.7	68
12	8.2	44.9	44.6	44.2	46.8	46.5	50.1	46.2	76
13	5.1	43.3	45.3	46.1	44.8	45.7	45.0	45.0	79
14	9.3	62.2	58.2	64.1	58.4	57.6	56.0	59.4	81
15	8.3	54.1	55.6	55.4	54.1	54.4	53.9	54.6	80

VO_{2rest} = oxygen consumption at rest; VO_2 10min-60min = oxygen consumption at respective time;
VO_{2mean} = mean oxygen consumption; %VO_{2peak} = percentage of peak oxygen consumption

Constant load test (repetition of highest workload below IAT)

IAT = individual anaerobic threshold

n	workload [W]	HR_{rest} [bpm]	HR 10min [bpm]	HR 20min [bpm]	HR 30min [bpm]	HR 40min [bpm]	HR 50min [bpm]	HR 60min [bpm]	HR_{mean} [bpm]	% HR_{max} [%]
1	300	68	150	153	152	153	155	157	153	83
2	250	64	150	156	162	164	172	171	163	82
3	250	67	148	151	152	156	158	157	154	76
4	250	71	141	143	148	150	151	150	147	79
5	250	56	144	145	145	149	153	151	148	79
6	300	51	149	146	152	146	153	154	150	80
7	300	62	157	162	162	164	168	166	163	91
8	250	60	140	143	145	146	146	148	145	77
9	200	68	152	154	155	159	164	161	158	84
10	300	74	168	172	178	179	180	178	176	97
11	250	50	149	155	158	163	164	163	159	81
12	200	72	144	150	153	157	161	164	155	81
13	200	94	182	186	191	195	197	197	191	88
14	300	72	156	165	167	170	171	172	167	90
15	250	76	151	155	160	163	165	166	160	84

HR_{rest} = heart rate at rest; HR 10min-60min = heart rate at respective time; HR_{mean} = mean heart rate
%HR_{max} = percentage of maximum heart rate

Constant load test (repetition of highest workload below IAT)

IAT = individual anaerobic threshold

n	BLa_{rest} [mmol·l^{-1}]	BLa 10min [mmol·l^{-1}]	BLa 20min [mmol·l^{-1}]	BLa 30min [mmol·l^{-1}]	BLa 40min [mmol·l^{-1}]	BLa 50min [mmol·l^{-1}]	BLa 60min [mmol·l^{-1}]	BLa_{mean} [mmol·l^{-1}]
1	0.5	2.0	2.0	1.9	1.8	1.6	1.8	1.9
2	1.5	3.5	2.9	2.4	2.5	2.2	5.0	3.1
3	1.1	1.5	1.7	1.3	1.3	1.1	1.1	1.3
4	0.5	2.7	2.2	3.3	3.1	1.4	1.6	2.4
5	1.2	0.8	0.9	0.9	0.9	0.9	1.3	0.9
6	0.8	1.7	1.5	1.3	1.3	1.1	1.4	1.4
7	1.4	5.1	5.4	4.6	5.5	3.8	3.2	4.6
8	1.7	2.4	1.7	1.6	1.7	1.2	1.2	1.6
9	1.4	2.5	2.5	2.8	2.6	3.2	3.2	2.8
10	1.3	5.2	5.9	6.0	7.5	8.8	8.3	6.9
11	0.5	1.0	1.4	0.9	1.2	1.1	0.9	1.1
12	1.5	2.6	2.6	2.6	2.6	2.4	2.6	2.6
13	1.1	4.8	5.7	6.0	6.4	6.7	7.1	6.1
14	1.5	2.6	3.1	3.4	3.2	3.0	2.9	3.0
15	1.0	1.6	1.8	2.3	2.1	2.2	1.9	2.0

BLa_{rest} = blood lactate concentration at rest; BLa 10min-60min = blood lactate concentration at respective time; BLa_{mean} = mean blood lactate concentration

Constant load test (repetition of highest workload below IAT)

IAT = individual anaerobic threshold

n	VO_{2rest} [ml·min^{-1}·kg^{-1}]	VO_2 10min [ml·min^{-1}·kg^{-1}]	VO_2 20min [ml·min^{-1}·kg^{-1}]	VO_2 30min [ml·min^{-1}·kg^{-1}]	VO_2 40min [ml·min^{-1}·kg^{-1}]	VO_2 50min [ml·min^{-1}·kg^{-1}]	VO_2 60min [ml·min^{-1}·kg^{-1}]	VO_{2mean} [ml·min^{-1}·kg^{-1}]	$\%VO_{2peak}$ [ml·min^{-1}·kg^{-1}]
1	7.5	52.6	54.1	51.7	53.0	47.8	52.8	52.0	77
2	7.5	39.1	40.6	40.1	41.0	43.4	42.8	41.2	73
3	6.2	50.8	51.1	50.6	51.5	51.4	50.5	51.0	71
4	9.3	45.3	44.4	45.7	45.4	45.0	45.5	45.2	76
5	6.4	47.1	47.3	46.5	48.0	47.2	49.6	47.6	73
6	9.5	51.2	52.0	50.5	53.3	52.6	53.5	52.2	78
7	6.9	52.2	56.5	55.0	51.4	52.1	51.0	53.0	80
8	7.0	46.6	46.3	32.1	47.7	46.6	47.5	44.5	59
9	8.4	44.9	44.2	45.4	48.5	47.6	46.3	46.2	68
10	10.5	59.2	59.2	60.0	61.6	61.9	59.9	60.3	89
11	6.9	57.5	58.8	58.7	57.5	59.4	59.0	58.5	73
12	7.9	41.7	40.3	40.0	44.4	41.4	43.6	41.9	69
13	5.3	40.1	40.2	42.1	40.9	42.7	41.1	41.2	73
14	7.6	64.3	59.2	58.2	59.3	60.4	59.5	60.1	82
15	7.5	45.2	48.8	52.2	50.1	49.8	51.5	49.6	73

VO_{2rest} = oxygen consumption at rest; VO_2 10min-60min = oxygen consumption at respective time; VO_{2mean} = mean oxygen consumption; $\%VO_{2peak}$ = percentage of peak oxygen consumption

Prediction of blood lactate (predictor: % HR_{max} = % heart rate reserve)

n	% HR_{max} [%]	$BLa_{observed}$ [mmol·l^{-1}]	$IV_{equation}$ [mmol·l^{-1}]	Δ $IV_{equation}$ - $BLa_{observed}$ [mmol·l^{-1}]	$GB_{equation}$ [mmol·l^{-1}]	Δ $GB_{equation}$ - $BLa_{observed}$ [mmol·l^{-1}]	SSD IV [mmol·l^{-1}]	SSD GB [mmol·l^{-1}]
1	83	1.9	1.6	-0.3	3.2	1.3	0.1	1.7
2	82	3.1	4.4	1.3	2.5	-0.5	1.8	0.3
3	76	1.3	1.8	0.5	2.2	0.9	0.2	0.8
4	79	2.4	3.1	0.7	2.9	0.6	0.6	0.3
5	79	0.9	0.9	-0.1	2.5	1.5	0.0	2.4
6	80	1.4	3.9	2.5	3.2	1.8	6.3	3.4
7	91	4.6	4.3	-0.3	3.6	-1.1	0.1	1.1
8	77	1.6	1.1	-0.6	1.0	-0.6	0.3	0.4
9	84	2.8	2.2	-0.6	1.8	-1.0	0.3	0.9
10	97	6.9	8.7	1.8	5.5	-1.4	3.1	2.0
11	81	1.1	2.8	1.7	2.5	1.4	2.9	2.0
12	81	2.6	1.7	-0.9	1.9	-0.6	0.8	0.4
13	88	6.1	4.0	-2.1	2.4	-3.7	4.5	13.7
14	90	3.0	2.8	-0.3	4.0	0.9	0.1	0.9
15	84	2.0	1.3	-0.7	2.4	0.4	0.5	0.2

predictor: %HR_{max} ; predicted variable: $BLa_{observerd}$ = blood lactate concentration observed during repetition test;
$IV_{equation}$ = individual regression equation; Δ $IV_{equation}$ - $BLa_{observed}$ = difference between predicted and observed BLa
$GB_{equation}$ = group-based regression equation; Δ $GB_{equation}$ - $BLa_{observed}$ = difference between predicted and observed BLa
SSD IV = sum of squared differences (individual regression); SSD GB = sum of squared differences (group-based regression)

Prediction of blood lactate (predictor: % HR_R = % heart rate reserve)

**n**	**% HR_R** [%]	**$BLa_{observed}$** [$mmol \cdot l^{-1}$]	**$IV_{equation}$** [$mmol \cdot l^{-1}$]	**Δ $IV_{equation}$ - $BLa_{observed}$** [$mmol \cdot l^{-1}$]	**$GB_{equation}$** [$mmol \cdot l^{-1}$]	**Δ $GB_{equation}$ - $BLa_{observed}$** [$mmol \cdot l^{-1}$]	**SSD IV** [$mmol \cdot l^{-1}$]	**SSD GB** [$mmol \cdot l^{-1}$]
1	76	1.9	1.0	-0.9	2.2	0.3	0.8	0.1
2	74	3.1	3.5	0.5	2.4	-0.7	0.2	0.5
3	79	1.3	1.6	0.2	1.0	-0.3	0.0	0.1
4	75	2.4	1.9	-0.5	1.4	-1.0	0.2	1.0
5	70	0.9	0.9	-0.1	2.0	1.0	0.0	1.0
6	75	1.4	1.9	0.6	3.8	2.4	0.3	5.8
7	90	4.6	3.5	-1.1	3.8	-0.8	1.2	0.7
8	63	1.6	1.7	0.0	1.9	0.3	0.0	0.1
9	79	2.8	2.9	0.1	2.3	-0.4	0.0	0.2
10	77	6.9	7.3	0.3	5.4	-1.6	0.1	2.4
11	80	1.1	1.4	0.4	2.2	1.2	0.1	1.4
12	65	2.6	2.0	-0.5	2.3	-0.3	0.3	0.1
13	88	6.1	5.0	-1.1	2.6	-3.5	1.3	12.1
14	94	3.0	3.1	0.1	3.2	0.2	0.0	0.0
15	71	2.0	1.4	-0.6	2.4	0.4	0.3	0.2

predictor: $\%HR_R$; predicted variable: $BLa_{observerd}$ = blood lactate concentration observed during repetition test;
$IV_{equation}$ = individual regression equation; Δ $IV_{equation}$ - $BLa_{observed}$ = difference between predicted and observed BLa
$GB_{equation}$ = group-based regression equation; Δ $GB_{equation}$ - $BLa_{observed}$ = difference between predicted and observed BLa
SSD IV = sum of squared differences (individual regression); SSD GB = sum of squared differences (group-based regression)

Prediction of blood lactate (predictor: % VO_{2peak} = % peak oxygen consumption)

**n**	**% VO_{2peak}** [%]	**$BLa_{observed}$** [$mmol \cdot l^{-1}$]	**$IV_{equation}$** [$mmol \cdot l^{-1}$]	**Δ $IV_{equation}$ - $BLa_{observed}$** [$mmol \cdot l^{-1}$]	**$GB_{equation}$** [$mmol \cdot l^{-1}$]	**Δ $GB_{equation}$ - $BLa_{observed}$** [$mmol \cdot l^{-1}$]	**SSD IV** [$mmol \cdot l^{-1}$]	**SSD GB** [$mmol \cdot l^{-1}$]
1	77	1.9	1.6	-0.3	3.2	1.3	0.1	1.7
2	73	3.1	4.4	1.3	2.5	-0.5	1.8	0.3
3	71	1.3	1.8	0.5	2.2	0.9	0.2	0.8
4	76	2.4	3.1	0.7	2.9	0.6	0.6	0.3
5	73	0.9	0.9	-0.1	2.5	1.5	0.0	2.4
6	78	1.4	3.9	2.5	3.2	1.8	6.3	3.4
7	80	4.6	4.3	-0.3	3.6	-1.1	0.1	1.1
8	59	1.6	1.1	-0.6	1.0	-0.6	0.3	0.4
9	68	2.8	2.2	-0.6	1.8	-1.0	0.3	0.9
10	89	6.9	8.7	1.8	5.5	-1.4	3.1	2.0
11	73	1.1	2.8	1.7	2.5	1.4	2.9	2.0
12	69	2.6	1.7	-0.9	1.9	-0.6	0.8	0.4
13	73	6.1	4.0	-2.1	2.4	-3.7	4.5	13.7
14	82	3.0	2.8	-0.3	4.0	0.9	0.1	0.9
15	73	2.0	1.3	-0.7	2.4	0.4	0.5	0.2

predictor: %VO_{2peak} ; predicted variable: $BLa_{observerd}$ = blood lactate concentration observed during repetition test;
$IV_{equation}$ = individual regression equation; Δ $IV_{equation}$ - $BLa_{observed}$ = difference between predicted and observed BLa
$GB_{equation}$ = group-based regression equation; Δ $GB_{equation}$ - $BLa_{observed}$ = difference between predicted and observed BLa
SSD IV = sum of squared differences (individual regression); SSD GB = sum of squared differences (group-based regression)

Prediction of blood lactate (predictor: % VO_{2R} = % oxygen consumption reserve)

n	% VO_{2R} [%]	$BLa_{observed}$ [mmol·l^{-1}]	$IV_{equation}$ [mmol·l^{-1}]	Δ $IV_{equation}$ - $BLa_{observed}$ [mmol·l^{-1}]	$GB_{equation}$ [mmol·l^{-1}]	Δ $GB_{equation}$ - $BLa_{observed}$ [mmol·l^{-1}]	SSD IV [mmol·l^{-1}]	SSD GB [mmol·l^{-1}]
1	72	1.9	1.6	-0.3	2.8	0.9	0.1	0.8
2	69	3.1	4.4	1.3	2.4	-0.7	1.8	0.5
3	69	1.3	1.8	0.5	2.3	1.0	0.2	1.0
4	72	2.4	3.1	0.7	2.7	0.3	0.6	0.1
5	70	0.9	0.9	-0.1	2.4	1.5	0.0	2.2
6	75	1.4	3.9	2.5	3.2	1.8	6.3	3.3
7	77	4.6	4.3	-0.3	3.5	-1.1	0.1	1.2
8	55	1.6	1.1	-0.6	1.1	-0.5	0.3	0.2
9	63	2.8	2.2	-0.6	1.7	-1.0	0.3	1.1
10	88	6.9	8.7	1.8	5.4	-1.5	3.1	2.2
11	71	1.1	2.8	1.7	2.6	1.5	2.9	2.2
12	65	2.6	1.7	-0.9	1.9	-0.6	0.8	0.4
13	69	6.1	4.0	-2.1	4.0	-2.1	4.5	4.4
14	80	3.0	2.8	-0.3	4.0	0.9	0.1	0.9
15	70	2.0	1.3	-0.7	2.5	0.5	0.5	0.2

predictor: %VO_{2R} ; predicted variable: $BLa_{observerd}$ = blood lactate concentration observed during repetition test;
$IV_{equation}$ = individual regression equation; Δ $IV_{equation}$ - $BLa_{observed}$ = difference between predicted and observed BLa
$GB_{equation}$ = group-based regression equation; Δ $GB_{equation}$ - $BLa_{observed}$ = difference between predicted and observed BLa
SSD IV = sum of squared differences (individual regression); SSD GB = sum of squared differences (group-based regression)

Constant load test (workload following IAT to confirm absence of MLSS)

IAT = individual anaerobic threshold

MLSS = maximum lactate steady state

**n**	**workload** [W]	**HR_{rest}** [bpm]	**HR 10min** [bpm]	**HR 20min** [bpm]	**HR 30min** [bpm]	**HR 40min** [bpm]	**HR 50min** [bpm]	**HR 60min** [bpm]	**HR_{peak}** [bpm]	**% HR_{max}** [%]
1	350	70	170	174	177				177	96
2	300	61	170	183					183	92
3	300	68	176	182	187				190	94
4	300	82	170	176					179	96
5	300	62	163	169					169	90
6	350	63	165	171	180				182	97
7	350	61	167	174					175	97
8	300	62	167						167	89
9	250	66	180						183	97
10	350	65	179						182	100
11	300	57	174	175	185				185	94
12	250	72	173						179	94
13	250	105	208						212	97
14	350	75	181						185	99
15	300	72	176	184	191				192	101

HR_{rest} = heart rate at rest; HR 10min-60min = heart rate at respective time; HR_{peak} = peak heart rate

$\%HR_{max}$ = percentage of maximum heart rate

Constant load test (workload following IAT to confirm absence of MLSS)

IAT = individual anaerobic threshold

MLSS = maximum lactate steady state

n	BLa_{rest} [mmol·l^{-1}]	BLa 10min [mmol·l^{-1}]	BLa 20min [mmol·l^{-1}]	BLa 30min [mmol·l^{-1}]	BLa 40min [mmol·l^{-1}]	BLa 50min [mmol·l^{-1}]	BLa 60min [mmol·l^{-1}]	BLa_{peak} [mmol·l^{-1}]
1	0.9	5.6	6.7	6.9				8.2
2	0.7	8.4	10.7					10.7
3	0.8	6.0	7.0	9.8				10.6
4	0.7	7.3	9.2					9.6
5	0.8	4.5	7.1					7.1
6	0.9	6.3	6.5	6.6				6.6
7	1.2	7.4	10.5					10.5
8	1.0	6.9						6.9
9	1.0	10.2						14.4
10	1.0	11.9						12.7
11	0.8	5.5	6.2	7.9				8.2
12	0.6	9.1						10.4
13	1.1	13.3						22.0
14	1.1	11.6						13.4
15	0.9	4.8	7.5	10.0				10.9

BLa_{rest} = blood lactate concentration at rest; BLa 10min-60min = blood lactate concentration at respective time;
BLa_{peak} = peak blood lactate concentration

Constant load test (workload following IAT to confirm absence of MLSS)

IAT = individual anaerobic threshold

MLSS = maximum lactate steady state

n	VO_{2rest} [ml·min^{-1}·kg^{-1}]	VO_2 10min [ml·min^{-1}·kg^{-1}]	VO_2 20min [ml·min^{-1}·kg^{-1}]	VO_2 30min [ml·min^{-1}·kg^{-1}]	VO_2 40min [ml·min^{-1}·kg^{-1}]	VO_2 50min [ml·min^{-1}·kg^{-1}]	VO_2 60min [ml·min^{-1}·kg^{-1}]	$\%VO_{2top}$ [ml·min^{-1}·kg^{-1}]	$\%VO_{2peak}$ [ml·min^{-1}·kg^{-1}]
1	9.1	60.8	65.8	57.8				65.8	97.6
2	5.0	44.2	39.9					44.2	78.9
3	9.7	54.0	55.4	53.1				55.4	77.5
4	8.7	52.1	45.6					45.6	76.8
5	9.1	60.5	60.7					60.7	93.4
6	5.9	58.2	58.9	57.0				58.9	87.9
7	6.4	64.7	65.7					65.7	98.8
8	7.2	62.0						62.0	81.6
9	7.9	57.6						57.6	85.1
10	7.6	64.5						64.5	95.3
11	7.8	68.2	66.0	67.4				68.2	85.4
12	8.9	54.8						54.8	90.1
13	4.9	52.2						52.2	92.1
14	7.3	71.1						71.1	96.9
15	6.8	57.7	58.3	58.8				58.8	86.0

VO_{2rest} = oxygen consumption at rest; VO_2 10min-60min = oxygen consumption at respective time;
VO_{2top} = highest oxygen consumption; $\%VO_{2peak}$ = percentage of peak oxygen consumption
