# Supplementary material for: Interindividual Variation in the Relationship of Different Intensity Markers—A Challenge for Targeted Training Prescriptions
Source: PLoS One. 2016 Oct 27;11(10):e0165010. doi: 10.1371/journal.pone.0165010 (PMC5082935; doi:10.1371/journal.pone.0165010)
Supplement: S1 Protocol — (PDF) [file pone.0165010.s002.pdf]

Trial study protocol approved by the local ethics committee

(Aerztekammer des Saarlandes, Saarbruecken, Germany)

Interindividual variation in the relationship of different intensity markers - a challenge for targeted training prescriptions

1. Formalities:

1.1 **Designation of operation:**
Prescribing physical exercise reliably and precisely – interindividual variability of strain indicators and training stimulus

1.2 **Name of the responsible project leader and the involved physicians:**
Dr. med. Anne Hecksteden

1.3 **Type and number of inspection authorities and names of physicians in the case of multi-center-studies**
N.a.

1.4 **Name and address of the sponsor**
N.a.

1.5 **Has there ever been such a request to another ethics committee ?**
No.

1.5.1 **To which ?**
N.a.

1.5.2 **Submission of the vote including the restrictions of this ethics committee including the potentially exchanged correspondance.**
N.a.

2. Description and scientific justification of the project

2.1 **Explanation of the objective**
The aim of prescribing a specific training intensity is to induce a defined training stimulus, which is interindividually comparable. Thus, a reliable “dosage“ of training interventions provides the basis for structured and output-driven training programs in health-related and competitive sport and is obviously of vital importance within the scope of scientific training studies. A common procedure is the derivation of training areas relative to a certain reference value (e.g. maximum heart rate, maximum oxygen consumption) [1-3]. Normally, these reference values are provided by the implementation of incremental or ramp exercise tests. A distinction can be drawn between

maximal and submaximal or cardio-circulatory and metabolic reference values, respectively [4]. In the context of a “defined training stimulus“ it is assumed that during typical endurance training the relationship between performance-related courses of indicators of different strain categories is interindividually constant. However, the validity of this assumption is doubtful [4-5].
Therefore, the aim of this study is to quantify the interindividual variability of practice-oriented strain indicators in endurance exercise. Intraindividual variability is estimated by conduction of a repetition test. The study design provides additional variance explanation by considering interindividual differences as well as selective comparisons in standard relative intensity prescriptions.

2.2 Description of the current state of knowledge (literature)

Background:
Changes in gene expression are a basic mechanism of training adaption. Disturbances of cellular homeostasis are the starting points of signaling processes and can therefore be regarded as “molecular training stimulus” [4, 6, 7]. Physical activity influences numerous aspects of the cellular homeostasis in the working muscles (ATP/AMP ratio [8], free radicals [9], calcium, pH, substrates of energy metabolism etc.) indirectly but also in the endothelium by increased blood flow [10]. Metabolic and cardio-circulatoric strain are considered as the essential categories, which are easily accessible by measuring blood lactate concentration and heart rate (-reserve) [4].

Derivation of hypothesis and study design:
Normally, training prescriptions are derived relative to a reference value, which is determined during an exercise test (e.g. maximum heart rate or maximum oxygen consumption) [1-3]. In the context of a “defined training stimulus“ it is assumed that during typical endurance training the relationship between performance-related courses of indicators of different strain categories is interindividually constant (e.g. 60% VO_{2max} correspond to 80% HR_{max}, 2 $mmol \cdot l^{-1}$ blood lactate concentration and the first decrease of ATP/AMP ratio). However, the validity of this assumption is doubtful [4-5]. Yet, this has been analyzed only by pointwise comparisons (e.g. endurance tests at fixed percentages of VO_{2max}). Nevertheless, this practical approach is associated with two essential limitations.
(1) Information about performance-related courses of the strain indicators during endurance exercise is missing (lactate performance curve)
(2) By using relative intensity prescriptions (e.g. $\%VO_{2max}$), random measurement error of the reference value will inevitably be transmitted to the setting of the constant load tests. This necessarily influences the observed interindividual variability of the strain indicators and impedes the quantification of true interindividual variability.

2.3 Results of the pharmacological-toxicological pre-examination (laboratory tests and animal experiments)

N.a.

2.4 Submission of the entire test plan

Design

A) Incremental exercise test. Determination of IAT and VO_{2peak}.
(Cycle-spiroergometry. Start at 50 W, increase by 50 W every 3 min)

B) 4 constant load tests [11] of 30 min in random order + 1 repetition test (single-blind; interval 48-96 hours)

- Absolute intensities identical to the steps of the incremental test;
- 1 step above IAT (confirmation of MLSS) and 3 steps below IAT
- Repetition of the test with the highest workload below the maximum lactate steady state (determination of intraindividual variability)

C) Results of the constant load tests are interpolated to generate "lactate performance curves"

Procedure of the tests:

Standardization: No substantial physical activity of at least 47 hours and dietary record 24 hours prior to testing (comparable carbohydrate-rich diet, sufficient liquid intake, no coffee, no alcohol). Same time of day for each subject (± 1 hour). Protocolling of temperature, humidity and usage of a ventilator in all tests.

Before inclusion: Medical history and physical examination, resting ECG, blood count, clinical chemistry

Incremental test: Exercise-ECG. Cycle-spiroergometry (Cyclus 2, measurement of heart rate and blood lactate concentration every 3 min, at exhaustion and 1, 3, 5, 7 und 10 min post-exercise)

Constant load test: Cycle-spiroergometry (Cyclus 2, measurement of heart rate and blood lactate concentration every 10 min)

Subjects:

Trained male cyclists aged between 18 and 35 years (VO_{2peak} ≥ 45 ml/kg/min; IAT > 200 W)
n=20

Pimary outcome measures: (constant load test)

Classic: Heart rate, blood lactate concentration, oxygen consumption

Secondary: Time-dependent changes in constant load tests, e.g. heart rate-drift, "classics" in incremental tests

Issue and analysis

Preliminary: Description of the lactate performance curves generated by results of constant load tests

1) Quantification of the interindividual variability between the curves (variance explanation)
- Sum of squared differences of individual curves vs. pooled data
- Random effects model
- Possibly exemplary pointwise comparisons

2) Comparison of different reference values (if applicable in combination)
- Regression model (or comparison of confident intervals)

2.5 **Envisaged overall duration of examination**
4 months.

2.6 **Justification for the need of studies on humans**
Particularly with regard to the interindividual variability of strain indicators it can be assumed, that generalisability to other species is not feasible. Therefore, examinations in humans are necessary to allow conclusions for training practice but also for application in scientific studies.

2.6.1 **Study on healthy subjects ?**
Yes, healthy male competitive athletes (cyclists).

2.6.2 **Study on patients ?**
No.

2.6.3. **Inclusion criteria**
Healthy male competitive athletes (cyclists) aged between 18 and 35 years. VO_{2peak} ≥ 45 ml/kg/min; IAT > 200 W.

2.6.4 **Exclusion criteria**
Every prolonged medication except a well-controlled substituted hypothyroidism, internal medical diseases representing contraindication for physical stress [12], orthopaedic diseases or injuries incompatible with physical stress. These criteria were evaluated by a physician within a physical examination prior to study enrolment.

2.6.5 **Intermediate exclusion criteria**
New onset of diseases representing contraindications for physical stress. Non-adherence to the study protocol.

2.6.6 **Concurrent medication**
Exclusion criteria.

2.6.7 Considering potential contraindications

All limitations of sport capability would represent contraindications for study enrolment. Contraindications for venous blood sampling are rare, unless they are incompatible with competitive activity.

2.6.8 Statement to possible risks and side effects (including the not yet described)

The risk of venous blood sampling is incorrect needle placement, haematoma formation and local inflammations (e.g. phlebitis). At this point it must be mentioned that blood sampling within a routine physical examination involves the same risks.

2.6.9 Discontinuation criteria

New onset (permanent or transient) of limitations of sport and training capability including acute infections.

2.7.0 Designation of advisory and control commission

N.a.

3 References:

1. Vanhees, L., et al., *Importance of characteristics and modalities of physical activity and exercise in the management of cardiovascular health in individuals with cardiovascular risk factors: recommendations from the EACPR. Part II.* Eur J Prev Cardiol, 2012. **19**(5): p. 1005-33.
2. Vanhees, L., et al., *Importance of characteristics and modalities of physical activity and exercise in defining the benefits to cardiovascular health within the general population: recommendations from the EACPR (Part I).* Eur J Prev Cardiol, 2012. **19**(4): p. 670-86.
3. Garber, C.E., et al., *American College of Sports Medicine position stand. Quantity and quality of exercise for developing and maintaining cardiorespiratory, musculoskeletal, and neuromotor fitness in apparently healthy adults: guidance for prescribing exercise.* Med Sci Sports Exerc, 2011. **43**(7): p. 1334-59.
4. Mann, T., et al., *Methods of prescribing relative exercise intensity: physiological and practical considerations.* Sports Med, 2013. **43**(7): p. 613-25.
5. Scharhag-Rosenberger, F., et al., *Exercise at given percentages of VO2max: heterogeneous metabolic responses between individuals.* J Sci Med Sport, 2010. **13**(1): p. 74-9.
6. Coffey, V.G., et al., *The molecular bases of training adaptation.* Sports Med, 2007. **37**(9): p. 737-63.
7. Hawley, J.A., et al., *Signalling mechanisms in skeletal muscle: role in substrate selection and muscle adaptation.* Essays Biochem, 2006. **42**: p. 1-12.
8. Akerstrom, T.C., et al., *Oral glucose ingestion attenuates exercise-induced activation of 5'-AMP-activated protein kinase in human skeletal muscle.* Biochem Biophys Res Commun, 2006. **342**(3): p. 949-55.
9. Abruzzo, P.M., et al., *Moderate exercise training induces ROS-related adaptations to skeletal muscles.* Int J Sports Med, 2013. **34**(8): p. 676-87.
10. Di Francescomarino, S., et al., *The effect of physical exercise on endothelial function.* Sports Med, 2009. **39**(10): p. 797-812.
11. Beneke, R., *Methodological aspects of maximal lactate steady state-implications for performance testing.* Eur J Appl Physiol, 2003. **89**(1): p. 95-9.
12. Kindermann, W., *Ergometrie-Empfehlungen für die ärztliche Praxis.* Dtsch Z Sportmed, 1987. **38**: p. 244-268.
