# Supplementary material for: Interindividual Variation in the Relationship of Different Intensity Markers—A Challenge for Targeted Training Prescriptions
Source: PLoS One. 2016 Oct 27;11(10):e0165010. doi: 10.1371/journal.pone.0165010 (PMC5082935; doi:10.1371/journal.pone.0165010)
Supplement: S2 Protocol — (PDF) [file pone.0165010.s003.pdf]

Studienprotokoll anerkannt durch die lokale Ethikkommission

(Aerztekammer des Saarlandes, Saarbruecken, Deutschland)

Interindividuelle Unterschiede im Verhältnis verschiedener Intensitätsmarker – eine Herausforderung für gezielte Trainingsvorgaben

1. Formales

1.1 Bezeichnung des Vorhabens
Körperliches Training verlässlich steuern – Interindividuelle Variabilität von Beanspruchungsindikatoren und Trainingsreiz

1.2 Name des verantwortlichen Leiters und seiner mitbetreuenden Ärzte
Dr. med. Anne Hecksteden

1.3 Art und Zahl der Prüfstellen und Namen der beteiligten Ärzte bei Multicenterstudien
Entfällt.

1.4 Name und Adresse des Sponsors
Entfällt.

1.5 Wurde schon bei einer anderen Ethikkommission ein diesbezüglicher Antrag gestellt ?
Nein.

1.5.1 Bei welcher ?
Entfällt.

1.5.2 Vorlage des Votums einschl. der von dieser Ethikkommission gemachten Auflagen einschl. des evtl. geführten Schriftwechsels
Entfällt.

2. Beschreibung und wissenschaftliche Begründung des Projekts

2.1 Erläuterung des Studienziels
Das Ziel von Intensitätsvorgaben im Training ist die Sicherstellung von definierten und interindividuell vergleichbaren Trainingsreizen. Eine solche verlässliche „Dosierung“ von Trainingsinterventionen bildet die Grundlage strukturierter und ergebnisorientierter Trainingsprogramme im Gesundheits- und Leistungssport und ist im Rahmen wissenschaftlicher Trainingsstudien offensichtlich von grundlegender Bedeutung. Ein übliches Vorgehen ist die Ableitung der Trainingsbereiche relativ zu einem Referenzwert (z.B. maximale Herzfrequenz oder maximale Sauerstoffaufnahme) [1-3]. Diese Referenzwerte

werden in der Regel im Rahmen eines Stufen- oder Rampentests ermittelt. Es lassen sich maximale und submaximale bzw. kardiozirkulatorische und metabolische Referenzwerte unterscheiden [4]. Vor dem Hintergrund des Ziels „definierter Trainingsreiz" wird dabei vorausgesetzt, dass die leistungsbezogenen Verläufe der Indikatoren verschiedener Beanspruchungskategorien bei trainingstypischen Dauerbelastungen in einem interindividuell konstanten Verhältnis stehen. Die Gültigkeit dieser Annahme erscheint jedoch fraglich [4-5].
Ziel der vorliegenden Studie ist es daher die interindividuelle Variabilität von praxisrelevanten Beanspruchungsindikatoren bei Dauerbelastungen zu quantifizieren. Die intraindividuelle Variabilität wird über einen Wiederholungstest berücksichtigt. Das Design der Studie ermöglicht dabei sowohl eine Aussage über die zusätzliche Varianzaufklärung durch eine Berücksichtigung der interindividuellen Unterschiede als auch punktuelle Vergleiche bei praxisüblichen, relativen Intensitätsvorgaben.

2.2 Darstellung des bisherigen Wissensstandes (Literatur)

Hintergrund:

Änderungen der Genexpression sind ein grundlegender Mechanismus von Trainingsanpassungen. Auslenkungen der (zellulären) Homöostase sind ein wesentlicher Ausgangspunkt der entsprechenden Signalwege und können daher als „molekularer Trainingsreiz" betrachtet werden [4, 6, 7]. Körperliche Aktivität beeinflusst eine Vielzahl von Aspekten der zellulären Homöostase in der arbeitenden Muskulatur (ATP/AMP-Quotient [8], freie Radikale [9], Calcium, pH, Substrate des Energiestoffwechsels etc.) mittelbar aber auch z.B. im Endothel durch den erhöhten Blutfluss [10]. Als wesentliche Kategorien gelten die metabolische und die kardiozirkulatorische Beanspruchung die über die Blutlaktatkonzentration bzw. die Herzfrequenz (-reserve) relativ leicht zugänglich sind [4].

Ableitung von Hypothese und Studiendesign:

In der Regel erfolgt die Ableitung von Trainingsvorgaben relativ zu einem mittels Belastungsuntersuchung ermittelten Referenzwert (z.B. maximale Herzfrequenz oder maximale Sauerstoffaufnahme) [1-3]. Vor dem Hintergrund des Ziels „definierter Trainingsreiz" wird dabei vorausgesetzt, dass die leistungsbezogenen Verläufe der Indikatoren verschiedener Beanspruchungskategorien bei trainingstypischen Dauerbelastungen in einem interindividuell konstanten Verhältnis stehen (z.B. 60 % VO_{2max} entsprechen 80 % HF_{max}, 2 mmol/l Laktat und einem ersten Abfall des ATP/AMP-Quotienten). Die Gültigkeit dieser Annahme erscheint jedoch fraglich [4-5]. Allerdings wurde dies bisher nur durch punktuelle Vergleiche untersucht (z.B. Dauertests bei ausgewählten Prozentsätzen der VO_{2max}). Diese praxisnahe Herangehensweise beinhaltet jedoch zwei wesentliche Limitationen:

(1) Es sind keine Aussagen über den belastungsabhängigen Verlauf der Beanspruchungsindikatoren im Dauertest möglich („Leistungskurven").

(2) Durch die Vorgabe relativer Intensitäten (z.B. %VO_{2max}) wird die zufällige Streuung bei der Bestimmung des Referenzwerts in die Belastungsvorgabe für den Dauertest übertragen. Dies beeinflusst notwendiger Weise die beobachtete interindividuelle Variabilität der Beanspruchungsindikatoren, was

die Quantifizierung der wahren interindividuellen Variabilität wesentlich erschwert.
Zudem handelt es sich bei den bisher untersuchten Parametern in Bezug zur Auslenkung der zellulären Homöostase lediglich um gut messbare Surrogatparameter. Deren quantitativer Zusammenhang mit den molekularen Prozessen die letztlich zu einer trainingsbedingt veränderten Genexpression in der Muskelzelle führen ist letztlich weitgehend unbekannt. Zusätzlich zu den gängigen Beanspruchungsindikatoren soll daher auch die hierdurch ausgelöste Aktivierung zentraler Signalwege in der Skelettmuskulatur erfasst werden.

2.3 Ergebnisse der pharmakologisch-toxikologischen Vorprüfungen (Labor- und Tierversuche)

Entfällt.

2.4 Vorlage des gesamten Prüfplans in deutscher Sprache

Design

A) Stufentest mit Bestimmung von IAS und VO_{2peak}
(Fahrrad-Spiroergometrie, Beginn bei 50 W, Steigerung alle 3 min um 50 W)

B) 4 Dauertests à 30 min [11] in randomisierter Reihenfolge + 1 Wiederholungstest
(einfach-blind; Abstand 48-96 Stunden)

- Absolute Belastungsvorgabe entsprechend der Stufen im Stufentest;
- Erste überschwellige Stufe (Bestätigung MLSS) und 3 darunter
- Wiederholung des höchsten unterschwelligen Tests (Bestimmung der intraindividuellen Variabilität

C) Erstellung von „Leistungskurven“ mit Interpolation der Dauertestergebnisse

Ablauf der Untersuchungstermine:

Standardisierung: Keine wesentliche körperliche Aktivität für mind. 47 Stunden, Ernährungsprotokoll in den 24 Stunden vor der Untersuchung (vergleichbare, kohlenhydratreiche Ernährung, reichlich trinken, kein Kaffee, kein Alkohol), Für jeden Probanden gleiche Tageszeit für alle Tests (± 1 Stunde). Protokollierung von Temperatur, Luftfeuchtigkeit, Trinkmenge und Ventilatorgebrauch bei allen Tests.

Vor Einschluss:	Anamnese und körperliche Untersuchung, Ruhe-EKG, Blutbild, Klinische Chemie
Stufentest:	Belastungs-EKG. Fahrrad-Spiroergometrie (Cyclus 2, Herzfrequenz und Laktat alle 3 Minuten, bei Abbruch und 1.,3.,5.,7. und 10. Minute der Nachbelastung)

<u>Dauertest:</u> Fahrrad-Spiroergometrie (Cyclus 2, Herzfrequenz und Laktat alle 10 Minuten)

<u>Probanden:</u>

Trainierte männliche Radsportler zwischen 18 und 35 Jahren (VO_{2peak} ≥ 45 ml/kg/min; IAS > 200 W)
n=20

<u>Primäre Zielparameter:</u> (Dauertest)

Klassiker: Herzfrequenz, Laktatkonzentration, Sauerstoffaufnahme

<u>Sekundär:</u> Zeitabhängige Veränderungen in Dauertests z.B. HF-Drift
„Klassiker" im Stufentest

<u>Fragestellungen und Analyse</u>

Vorbereitend: Beschreibung der Leistungskurven aus Dauertests

1) Quantifizierung der interindividuellen Variabilität der Verläufe (Varianzbeitrag)
- Summe quadrierter Residuen in Einzelverläufen vs. gepoolten Daten
- Random effects-Modell
- Evtl. Beispielhafte punktuelle Vergleiche

2) Vergleich verschiedener Referenzwerte (ggf. in Kombination)
- Regressionsmodell (oder Vergleich von Konfidenzintervallen)

2.5 Vorgesehene Gesamtdauer der Untersuchungen
4 Monate.

2.6 Begründung der Notwendigkeit von Studien an Menschen
Gerade bei der interindividuellen Variabilität von Beanspruchungsmarkern ist davon auszugehen, dass eine Generalisierung auf andere Spezies nicht möglich ist. Um Schlussfolgerungen für die Trainingspraxis aber auch für die Anwendung in wissenschaftlichen Studien zu ermöglichen, ist daher eine Untersuchung am Menschen notwendig.

2.6.1 Studie an gesunden Probanden ?
Ja, gesunde, leistungssportlich aktive Männer (Radsportler).

2.6.2 Studie an Patienten ?
Nein.

2.6.3. Einschlusskriterien
Gesunde, leistungssportlich aktive männliche Radsportler zwischen 18 und 35 Jahren. VO_{2peak} ≥ 45 ml/kg/min; IAS > 200 W.

2.6.4 Ausschlusskriterien
Jedwede Dauermedikation mit Ausnahme einer stabil eingestellten Substitution bei Hypothyreose, internistische Erkrankungen, die eine Kontraindikation für sportliche Belastungen darstellen [1], orthopädische Erkrankungen oder Verletzungen, die eine sportliche Belastung unmöglich machen. Diese Kriterien werden im Rahmen einer ärztlichen Untersuchung vor Einschluss in die Studie überprüft.

2.6.5 Zwischenausschlusskriterien
Neuauftreten von Erkrankungen, die eine Kontraindikation für sportliche Belastungen darstellen. Mangelnde Compliance mit den Studienvorgaben.

2.6.6 Begleitmedikation
Auschlusskriterium.

2.6.7 Eingehen auf etwaige Kontraindikationen
Sämtliche Einschränkungen der Sporttauglichkeit würden Kontraindikationen für die Aufnahme in die Studie darstellen. Kontraindikationen für die Muskelbiopsie werden durch die ärztliche und laborchemische Untersuchung vor Einschluss in die Studie ausgeschlossen. Kontraindikationen für venöse Blutentnahmen existieren kaum oder sind nicht mit leistungssportlicher Aktivität zu vereinbaren.

2.6.8 Stellungnahme zu möglichen, auch bisher noch nicht beschriebenen Risiken und Nebenwirkungen
Das Risiko venöser Blutentnahmen besteht in Fehlpunktion, Hämatombildung und lokalen Entzündungen (z. B. Phlebitis). An dieser Stelle ist zu erwähnen, dass eine Blutentnahme bei einer routinemäßigen Gesundheitsuntersuchung dieselben Risiken birgt.

2.6.9 Abbruchkriterien
Neuauftretende (dauerhafte oder vorübergehende) Einschränkungen der Sporttauglichkeit und Trainingsfähigkeit einschließlich akuter Infekte.

2.7.0 Nennung der Begleit- und Kontrollkommission.
Entfällt.

3 Referenzen:

1. Vanhees, L., et al., *Importance of characteristics and modalities of physical activity and exercise in the management of cardiovascular health in individuals with cardiovascular risk factors: recommendations from the EACPR. Part II.* Eur J Prev Cardiol, 2012. **19**(5): p. 1005-33.
2. Vanhees, L., et al., *Importance of characteristics and modalities of physical activity and exercise in defining the benefits to cardiovascular health within the general population: recommendations from the EACPR (Part I).* Eur J Prev Cardiol, 2012. **19**(4): p. 670-86.
3. Garber, C.E., et al., *American College of Sports Medicine position stand. Quantity and quality of exercise for developing and maintaining cardiorespiratory, musculoskeletal, and neuromotor fitness in apparently healthy adults: guidance for prescribing exercise.* Med Sci Sports Exerc, 2011. **43**(7): p. 1334-59.
4. Mann, T., et al., *Methods of prescribing relative exercise intensity: physiological and practical considerations.* Sports Med, 2013. **43**(7): p. 613-25.
5. Scharhag-Rosenberger, F., et al., *Exercise at given percentages of VO2max: heterogeneous metabolic responses between individuals.* J Sci Med Sport, 2010. **13**(1): p. 74-9.
6. Coffey, V.G., et al., *The molecular bases of training adaptation.* Sports Med, 2007. **37**(9): p. 737-63.
7. Hawley, J.A., et al., *Signalling mechanisms in skeletal muscle: role in substrate selection and muscle adaptation.* Essays Biochem, 2006. **42**: p. 1-12.
8. Akerstrom, T.C., et al., *Oral glucose ingestion attenuates exercise-induced activation of 5'-AMP-activated protein kinase in human skeletal muscle.* Biochem Biophys Res Commun, 2006. **342**(3): p. 949-55.
9. Abruzzo, P.M., et al., *Moderate exercise training induces ROS-related adaptations to skeletal muscles.* Int J Sports Med, 2013. **34**(8): p. 676-87.
10. Di Francescomarino, S., et al., *The effect of physical exercise on endothelial function.* Sports Med, 2009. **39**(10): p. 797-812.
11. Beneke, R., *Methodological aspects of maximal lactate steady state-implications for performance testing.* Eur J Appl Physiol, 2003. **89**(1): p. 95-9.
12. Kindermann, W., *Ergometrie-Empfehlungen für die ärztliche Praxis.* Dtsch Z Sportmed, 1987. **38**: p. 244-268.
